# Supplementary figures and images for: A special satellite-like RNA of a novel hypovirus from Pestalotiopsis fici broadens the definition of fungal satellite
Source: PLoS Pathog. 2023 Jun 7;19(6):e1010889. doi: 10.1371/journal.ppat.1010889 (PMC10281576; doi:10.1371/journal.ppat.1010889)

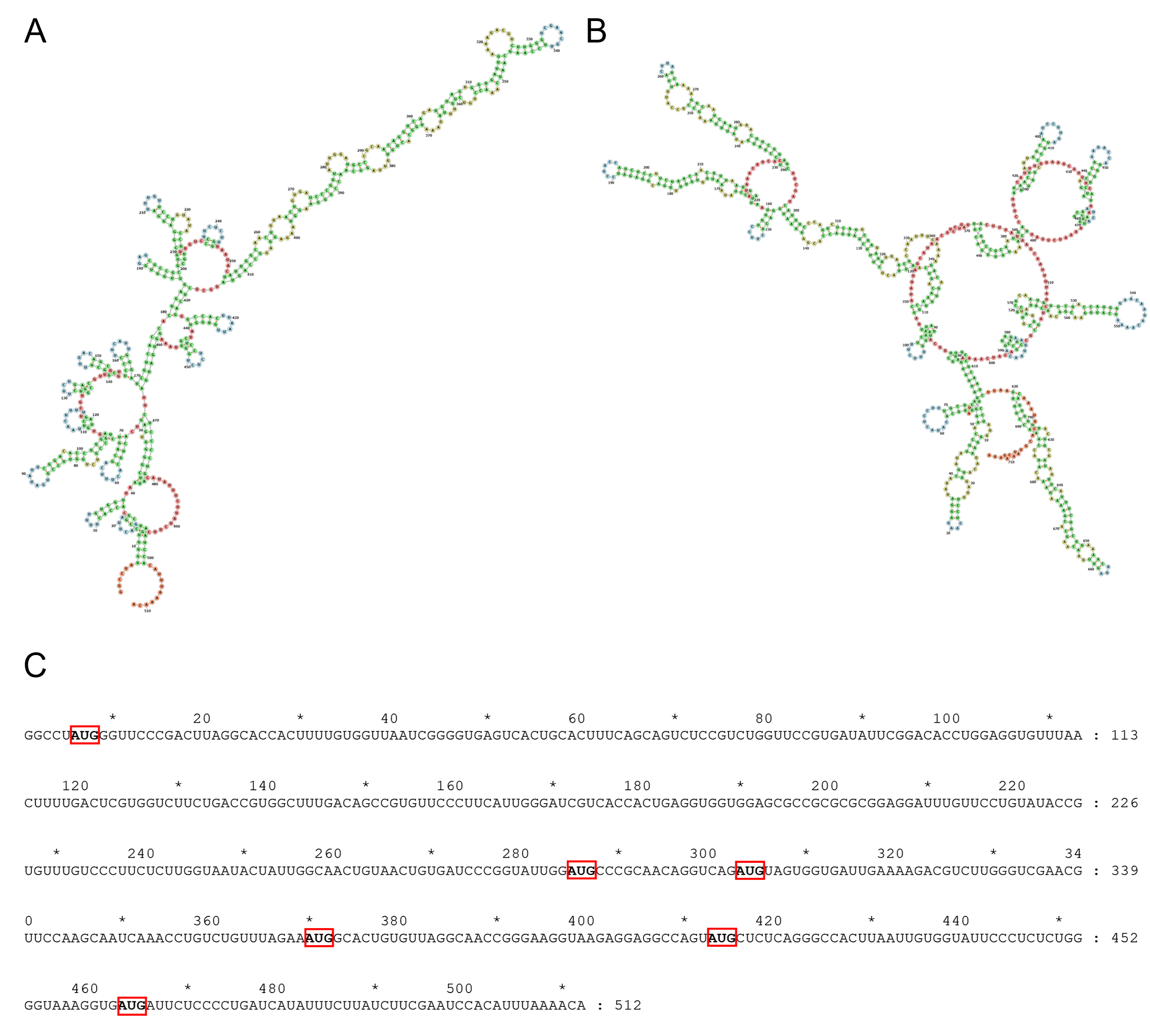

Supplement: S1 Fig — (A and B) The secondary structures of 5′- and 3′-UTR of PfHV1 were predicted in RNAfold, respectively. (C) Six AUGs in the 5′-UTR of PfHV1 were found. (TIF) [file ppat.1010889.s004.tif]

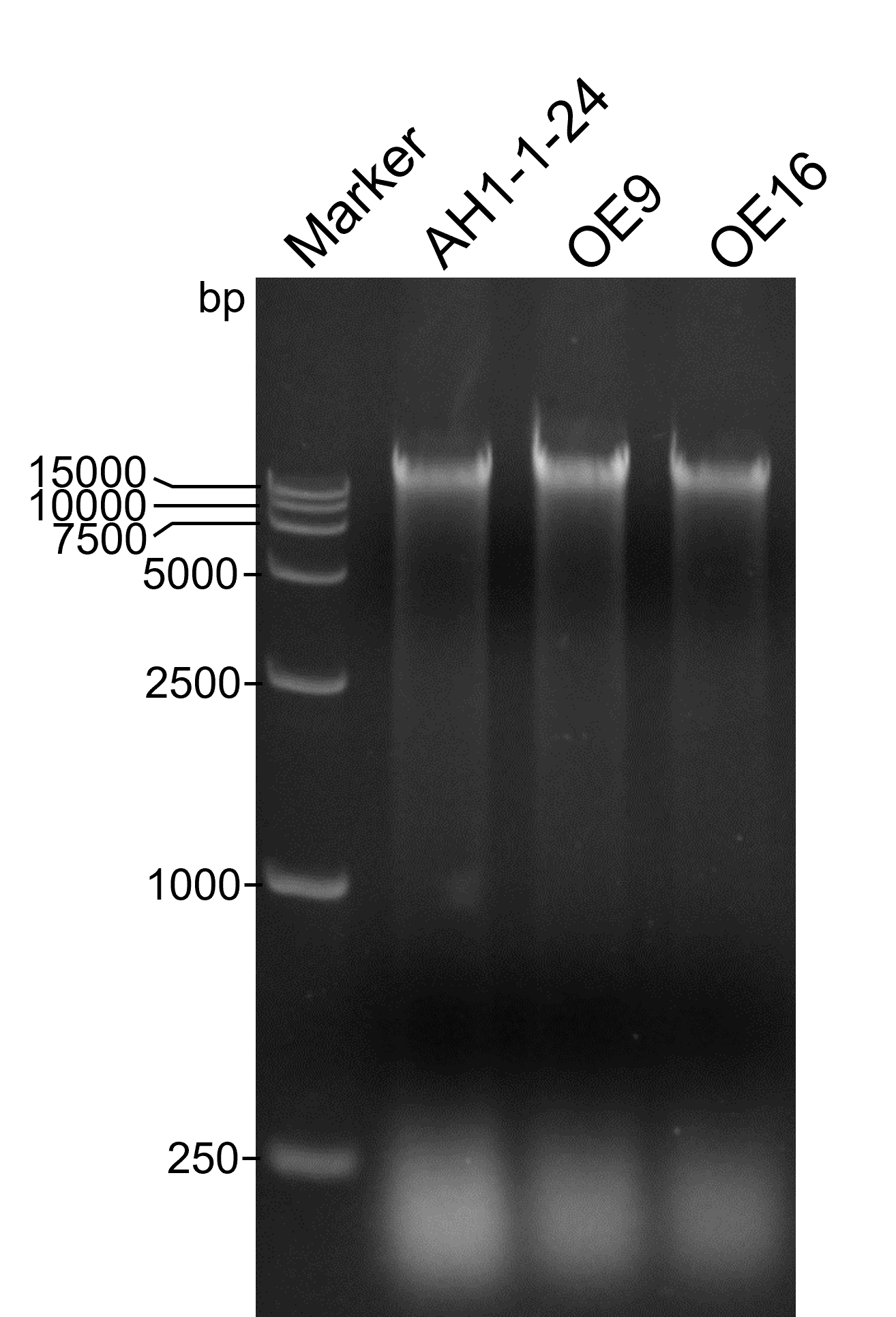

Supplement: S2 Fig — (TIF) [file ppat.1010889.s005.tif]

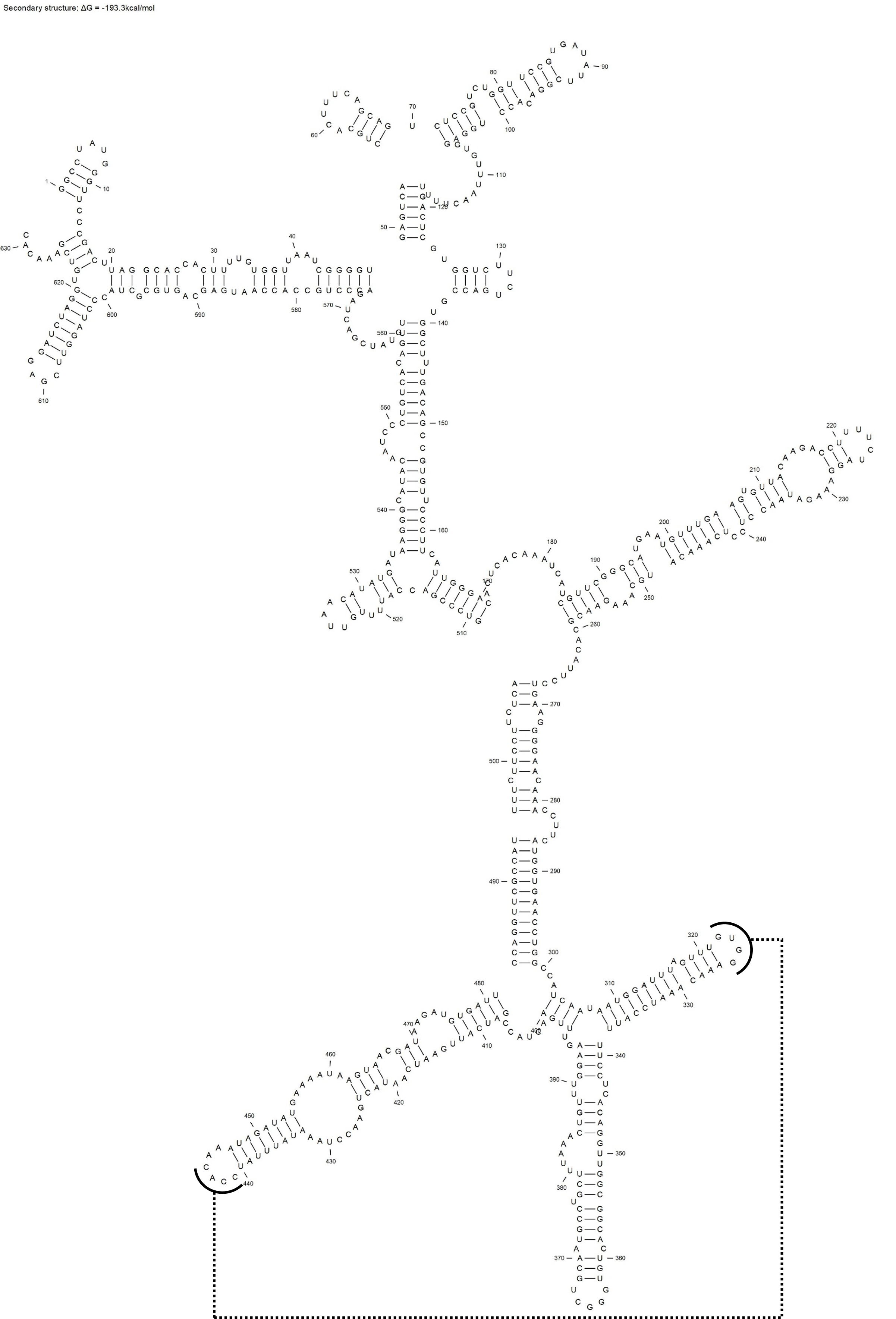

Supplement: S3 Fig — Two black curves refer to the nucleotides that make up a potential pseudoknot. (TIF) [file ppat.1010889.s006.tif]
